# Challenges of the Effectiveness of Traumatic Brain Injuries Biomarkers in the Sports-Related Context

**DOI:** 10.3390/jcm12072563

**Published:** 2023-03-29

**Authors:** Rossella Tomaiuolo, Martina Zibetti, Chiara Di Resta, Giuseppe Banfi

**Affiliations:** 1 Faculty of Medicine, Università Vita-Salute San Raffaele, 20132 Milan, Italy; 2 IRCCS Galeazzi-Sant’Ambrogio, 20157 Milan, Italy

**Keywords:** traumatic brain injury (TBI), sports-related TBI, brain injury markers/biomarkers, TBI biomarker, outcome assessment

## Abstract

Traumatic brain injury affects 69 million people every year. One of the main limitations in managing TBI patients is the lack of univocal diagnostic criteria, including the absence of standardized assessment methods and guidelines. Computerized axial tomography is the first-choice examination, despite the limited prevalence of positivity; moreover, its performance is undesirable due to the risk of radiological exposure, prolonged stay in emergency departments, inefficient use of resources, high cost, and complexity. Furthermore, immediacy and accuracy in diagnosis and management of TBIs are critically unmet medical needs. Especially in the context of sports-associated TBI, there is a strong need for prognostic indicators to help diagnose and identify at-risk subjects to avoid their returning to play while the brain is still highly vulnerable. Fluid biomarkers may emerge as new prognostic indicators to develop more accurate prediction models, improving risk stratification and clinical decision making. This review describes the current understanding of the cellular sources, temporal profile, and potential utility of leading and emerging blood-based protein biomarkers of TBI; its focus is on biomarkers that could improve the management of mild TBI cases and can be measured readily and directly in the field, as in the case of sports-related contexts.

## 1. Introduction

Every year, 69 million people worldwide suffer from traumatic brain injury (TBI) [1], defined by the WHO Neurotrauma Task Force as a blow to the head that causes acute impairment of brain function. Higher rates of TBI are seen in the adult population over 75, in children under 5, and in adolescents between 15 and 24 years of age [2].

TBI is a high-risk condition with a significant public health and socioeconomic impact because of its high mortality rate, morbidity, and disability rate [1].

The diagnosis of TBI is usually made in the emergency room using the Glasgow Coma Scale (GCS), universally recognized in the classification of TBI. The GCS allows classification of patients into three presumptively homogeneous groups for risk of post-traumatic cerebral hematoma, need for neurosurgery, and prognosis: severe head injury (GCS 3–8), moderate head injury (GCS 9–13), and mild head injury (GCS 14–15) [3].

Computed axial tomography (CT) is the leading method of radiological examination [4], it is estimated to have a sensitivity of 100%, with a positive predictive value of 10%, a negative predictive value of 100%, and a specificity of 51% [5]. MRI can also be used to assess traumatic brain injuries; it is more sensitive than CT in identifying the precise location and extent of intracranial haemorrhage and associated edema [6]. Nevertheless, conventional CT remains the initial imaging modality of choice since it is more available and cost effective, it requires shorter imaging time, and it is easier to perform [7]. However, the systematic carrying out of CT scanning in all patients is undesirable due to the limited prevalence of positivity, radiological risk of exposure, extended stay in emergency departments, inefficient use of resources, and its high cost and complexity [4].

Altogether, 90% of brain injuries are classified as mild TBI (mTBI) [8], about 15 times more frequent than moderate and over 20 times more than severe [3]. The main manifestations of this trauma are a brief loss of consciousness (less than 30 min), confusion, or post-traumatic amnesia not attributable to other factors, such as psychological trauma or intoxication from alcohol or drugs [2].

The Mild TBI Committee of the American Congress of Rehabilitation Medicine, revised by the World Health Organization (WHO), declared that “mTBI is defined by a Glasgow Coma Scale score between 13 and 15 at 30 min post-injury, and one or more of the following symptoms: <30 min loss of consciousness; <24 h post-traumatic amnesia (PTA); impaired mental state at the time of the accident (confusion, disorientation, etc.); and/or transient neurological deficit” [9].

It is essential to consider that mTBI can sometimes be associated with severe intracranial injuries: the patient can face the risk of complications, in particular intracranial bleeding, at various times: immediately up to about 48–72 h, in the medium term up to 4–8 weeks, and in the long term up to a few months. For this reason, risk stratification is essential for diagnosis based on integrating clinical anamnestic data, trauma (dynamics) data, and drug history [10]. mTBI may also give rise to neurodegenerative disorders and increase survivors’ risk of developing chronic behavioral and neurological impairment affecting the quality of their lives [11]. mTBIs are commonly observed in collision sports athletes and military personnel, and can also result from falls, motor vehicle accidents, or assaults [12].

Exposure to repeated mTBIs may lead to debilitating long-term neurological conditions: When the brain has not fully recovered from previous trauma, it can be highly vulnerable to repeated mTBIs. There is a significant clinical need for objective tests that could help to diagnose mTBIs and identify ‘‘at-risk’’ people. The clinical management of mTBI must be based on the probability of developing neurological complications (evolutionary risk), considering the absence or presence of one or more pre-existing or consequent risk factors [4]. In addition to the primary injury that arises immediately after the trauma, there may be secondary injuries resulting in a series of molecular and cellular reactions that last for a long time after the trauma, leading to neuronal and astroglia injuries, axonal disruption, and inflammation [11].

There is a strong need for prognostic indicators of long-term outcomes following mTBI, to identify at-risk subjects to avoid their return to play or duty while the brain is still highly vulnerable. Fluid biomarkers may emerge as new prognostic indicators to develop more accurate prediction models, improving risk stratification and clinical decision-making [12,13]. Much progress has been made in understanding the cellular sources, temporal profiles, and potential utility of key and emerging blood protein biomarkers.

This review focuses on biomarkers that may improve the assessment of mild TBI in clinical and field settings, particularly as prognostic factors in sports-related TBI. The focus is on the markers’ characteristics, the methodological aspects, and the technological development of the detection devices.

## 2. Methods

In order to identify sports-related mTBI biomarkers and to assess the feasibility of their introduction in clinics to improve patient management, a comparison of guidelines for TBI patient management was carried out and a literature review was conducted focusing on biomarkers released after TBI.

The consulted guidelines were the “Canadian CT Head Rule”, the “Scandinavian Guidelines”, the “New Orleans Criteria for TC scan in mild head injury”, the “guidelines of the National Institute for Health and Care Excellence” (NICE 2014), and those of the Neurotraumatology Committee of the World Federation of Neurosurgical Societies (NCWFS).

The search for bibliographic sources regarding TBI biomarkers was carried out on PubMed, Science Direct, and Cochrane Library for indexed articles in English. The following keywords were used: “brain injury markers”, “brain injury biomarkers”, “neurodegeneration biomarkers”, “TBI biomarker”, and “TBI markers”.

The bibliographic sources for the analysis of the methodological assessment of TBI biomarkers and the technological development stage of biomarker detection devices were found by searching PubMed with the following keywords: “methods detection TBI biomarkers”, “TBI biomarkers detection devices”, “electrochemical sensors TBI”, “TBI biomarkers measurement methods”, and “optical detection TBI”.

The temporal window for all the research was fixed between 2012 and the current date, to focus on the most recent publications in the field. Manual searches for older but significant references cited in the reviewed articles were made when appropriate. All the articles included in this literature review were published in journals with an impact factor higher than 4.

## 3. Results

### 3.1. Current Guidelines

There is no uniformity concerning the guidelines to be followed nationally and internationally. There are significant between-center variations in policies for diagnostics, admission, and discharge decisions in patients with TBI in the emergency department and hospital ward [14].

Commonalities between the different guidelines include the focus of evaluation mainly on assessing the patient’s mental status, cranial nerves, sensory awareness, motor functions, and reflexes. Patients receiving antiplatelet/anticoagulant therapy should have treatment suspended for the entire duration of the observation [14]. Neurological imaging is essential to identify a patient with head trauma caused by acute injury or persistent symptoms, and computed tomography is the primary method of radiological examination [4].

Each country has its directives for CT use; for instance, in Europe and Canada, CT for minor head injury cases is used very selectively. In Italy in particular, CT is only recommended if a fracture has been shown by skull radiography; in Denmark, it is rarely ordered and then only by a neurosurgeon; in the UK and Spain, CT is only recommended for cases with a documented skull fracture, focal neurological deficit, or deterioration in mental status [15].

The most frequently used guidelines are the Canadian CT Head Rule, the Scandinavian Guidelines, the New Orleans Criteria for TC scan in mild head injury, the guidelines of the National Institute for Health and Care Excellence (NICE 2014), and those of the Neurotramatology Committee of the World Federation of Neurosurgical Societies (NCWFS) [14]. These guidelines differ in terms of the parameters taken into consideration, as shown in Table 1: The Canadian CT rules are based on five high-risk and two medium-risk criteria [15]; the Scandinavian guidelines also consider the serum levels of S100 calcium-binding protein B (S100B) [16,17]; the New Orleans Criteria for TC scan (NOC) included seven items and were only developed for use in patients with a GCS score of 15 [18]; the guidelines of the National Institute for Health and Care Excellence (NICE 2014) are based upon the Canadian CT head rule and lead to more CT scans being performed, but fewer skull radiographs and admissions [19,20,21]; and lastly, the Neurotraumatology Committee of the World Federation of Neurosurgical Societies (NCWFS) protocol is similar to the NICE guidelines. However, it is less strict and leads to more CTs [22,23].

### 3.2. Markers

An ideal biomarker should be easy to measure in accessible bodily fluids such as cerebrospinal fluid or blood (serum/plasma); it should allow repeated detection for monitoring the initial brain injury in the hours that follow, and its elevated levels should correlate directly with the presence of brain trauma and the degree of severity of traumatic brain injury. Therefore, all the substances that can be released because of neuronal cell injury, glial cell injury, axonal injury, and inflammation are potential biomarkers for TBI.

Research into blood-based TBI biomarkers has accelerated rapidly in the past decade, leading to the identification of proteins resulting from axonal, neuronal, or glial cell injuries and released into the interstitial fluid (ISF), cerebrospinal fluid (CSF), and blood circulation due to altered function of the blood–brain barrier (BBB) after TBI [12].

Neuroimaging techniques such as computed tomography (CT) and magnetic resonance imaging (MRI) can identify gross head injuries but not minute neural and structural changes typical of mild TBI. Conversely, fluid biomarkers are accurate tools that can be used to assess mild TBI pathophysiology [11]: increased understanding of individual biomarker trajectories in the hours, days, and weeks post-injury will enable a greater understanding of diagnostic windows from acute and chronic perspectives. It will furthermore allow studies to investigate the best potential biomarkers for predicting outcomes and tracking pathophysiological recovery or measuring response to treatment in mTBI clinical trials [12].

**Figure 1:**
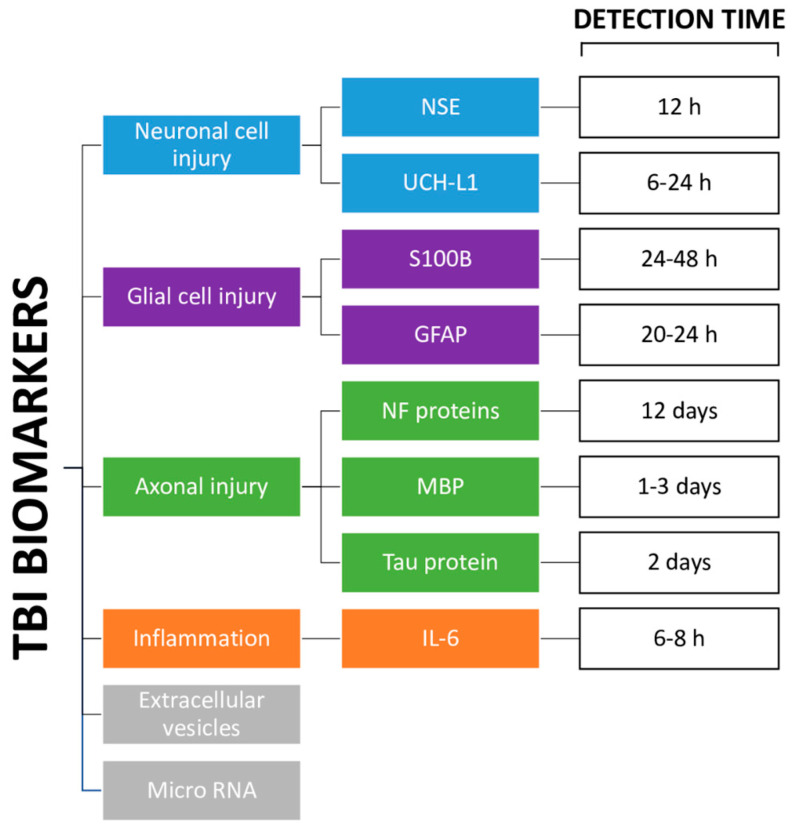
Diagram of the different types of TBI biomarkers. NSE: neuron-specific enolase [8]; UCH-L1: ubiquitin C-terminal hydrolase-L1 [8]; S100B: S100 calcium-binding protein B [9]; GFAP: glial fibrillary acidic protein [23]; NF: neurofilament proteins [9,21]; MBP: myelin basic protein [8,22]; Tau protein [8]; IL-6: interleukin-6 [25].

The following paragraphs report the current understanding of the cellular sources, temporal profile, and potential utility of leading and emerging blood-based protein biomarkers of TBI (Figure 1). Attention is focused on the characteristics that may favor use of these markers as surrogates for imaging techniques in the case of sports-related traumatic brain injuries.

#### 3.2.1. Markers of Neuronal Cell Body Injury

Neuron-specific enolase (NSE) is a neuronal cytoplasmatic enzyme necessary for the glycolytic pathway. NSE serum concentration rises in the first 12 h after TBI and declines within hours or days. The main drawback of using NSE as a TBI diagnostic tool is its high erythrocyte concentration. Therefore, it can also be elevated without TBI, for instance, in hemolysis or multi-trauma conditions [11]. Hemolysis of blood samples, extracranial injury, and physical exercise may generate false positives [12].

Ubiquitin C-terminal hydrolase-L1 (UCH-L1) is a neuronal cytoplasmatic deubiquitinating enzyme needed to remove abnormal neuronal proteins in physiological and pathological conditions. The increased serum concentration of UCH-L1 increases within the first 6–24 h after TBI and correlates with injury severity and clinical outcomes, including GCS score at admission and CT lesions [11]. In CT-positive patients, serum levels 6–12 h post-injury were greater in those with unfavorable neurological outcomes [12]. It is, therefore, a potential prognostic and diagnostic biomarker for mild, moderate, and severe TBIs.

#### 3.2.2. Markers of Glial Cell Injury

S100 calcium-binding protein B (S100B) is a calcium-binding protein within astroglial cells, which can be released in the extracellular space following trauma and ischemic events. Studies of moderate-to-severe TBI show peaks of S100B serum around 24–48 h after injury; however, a recent study found elevated levels at 1 h but not at 12 or 36 h post-concussion [12]. High levels of S100B are related to injury severity and predict the occurrence of post-concussion syndrome after mild TBI, poor clinical outcomes, and increased mortality. S100B protein is currently used in the early control of minimal, mild, and moderate TBI in Scandinavia, according to their head injury management guidelines (2007), to predict normal CT after mTBI, reducing unnecessary CT scans when S100B < 0.1 μg/L [11]. A limitation in using S100B as a prognostic biomarker after TBI is the extra-neural injury release from cardiac muscle, adipose tissue, and skeletal muscles. S100B is also present in melanocytes, and patients with darker skin show higher levels of the biomarker. Nonetheless, strenuous exercise and extracranial injury can increase blood S100B levels, thus potentially reducing their utility as a biomarker in sports-related concussions and polytrauma. Another pitfall relating to S100B is its short half-life of 90 min, making it difficult to use as a biomarker for brain injury [12,24,25].

Glial fibrillary acidic protein (GFAP) is an intermediate filament within astroglial cells that is needed to maintain their structure and to activate glial cells. After TBI, astroglial cells are activated and induce gliosis or glial scar formation, increasing the expression of GFAP [11]. There is a positive correlation between GFAP levels and TBI severity; therefore, GFAP can be used to assess mTBI severity and evaluate the need for neuroimaging with CT and MRI, predicting poor outcomes and the risk of developing cognitive and psychiatric disabilities [11]. Blood levels of GFAP peak within the first 24–48 h after mTBI, and the acute measures of blood GFAP in isolation or combined with UCH-L1 are susceptible to intracranial lesions in mTBI patients: this combination was recently approved by the FDA to reduce radiation exposure by CT [26]. Elevated UCH-L1 and GFAP measured within 12 h of injury indicate intracranial lesions requiring CT [12].

#### 3.2.3. Markers of Axonal Injury

Neurofilament proteins (NFs) are the primary components of the neuronal cytoskeleton. Phosphorylated filaments interact with each other in order to increase neuronal stability. However, after TBI, there is an increase in intracellular calcium levels that activate various calcium-dependent enzymes such as proteases, calpains, and phosphatase calcineurin, leading to NFs dephosphorylation, proteolysis, dissociation, and release in the extracellular space, then to CSF and blood [11]. NFs are formed by three different polypeptide subunits: light (NF-L, 68 kDa), medium (NF-M, 160 kDa), and heavy (NF-H, 200 kDa) [27]. Evidence suggests that NF levels, and in particular NF-L levels, rise throughout the first few weeks (12 d) post-injury, accurately distinguishing patients with TBI from controls up to six months post-injury and, even more impressively, between patients with mild, moderate, and severe TBI at 30 d post-injury [12,24]. NFs are specific for neurons and axons and are not affected by body trauma or strenuous physical activity; therefore, their extra-neural detection indicates neural death and axonal disintegration and lasts for days after the trauma, predicting poor outcomes, CT lesions, and the occurrence of chronic morbidities and cognitive disability [11,25].

Myelin basic protein (MBP) is a oligodendroglial protein released in the blood following axonal damage; it is not specific for CNS injury, because peripheral nerve injury also increases MBP blood levels. Its release is delayed (1–3 days after injury), and the initial levels do not correlate with the GCS. MBP would be an inaccurate diagnostic and prognostic biomarker, unsuitable for emergency room screenings [11,25].

Tau protein is a microtubule-associated protein (MAP) expressed mainly in the neurons to stabilize axonal microtubules. Within the context of TBI, microtubules release tau in response to mechanical stress, proteolytic cleavage by calpains and caspases, and calcium-dependent protein kinase activation, resulting in decreased microtubule binding and increased tau phosphorylation [24]. TBI increases tau release in CSF, and CSF tau concentration positively correlates with TBI severity and poor outcomes. Serum tau protein peaks only two days after TBI, reflecting injury severity and predicting the clinical outcome. Nevertheless, some studies reported that serum tau does not correlate with CT lesions and cannot predict post-concussion syndrome. CSF tau is, therefore, a more accurate diagnostic and prognostic tool than serum tau [11]. However, the release of tau from extracranial sources and after physical activity may limit the utility of tau in the context of sports-related mTBI. In addition to measures of total tau, quantification of tau in its phosphorylated form (p-tau) has also recently shown encouraging results as an acute marker indicator of mTBI, with elevated plasma levels found within 24 h of injury [12].

#### 3.2.4. Markers of Inflammation

Inflammation-associated proteins can function as blood mTBI biomarkers, becuase mTBI pathobiology is characterized by glial activation and release of proinflammatory cytokines [12]. Circulating cytokine changes appear to be restricted to the first few hours post-mTBI: interleukin-6 (IL-6) levels appear to be elevated within the first 6–8 h but return to control levels by 24–48 h after mTBI. Moreover, IL-6 was associated with CT and MRI findings and longer duration of symptoms after mTBI [28]. Interestingly, the temporal profile of IL-6 points to a distinctly different inflammatory profile in sports-related concussion (SRC) and military concussion versus the general unselected population with mTBI; athletes show early acute elevation (<8 h) with a return to baseline within 48 h, highlighting an earlier resolution of the inflammatory response in comparison with the general unselected population showing alterations lasting up to six months after injury. An explanation could be that the inflammatory response is milder in the relatively young and healthy athlete population than in the average mTBI patient [28,29,30]. Moreover, blood IL-1 receptor antagonist levels increase in the first few hours post-mTBI, remaining elevated for 24–48 h [29]. Other cytokines such as IL-8, IL-10, and TNF-α are excessively produced after TBI, but their correlation with injury severity and outcomes is yet to be confirmed; the best biomarker for mTBI is, therefore, IL-6 [11].

It should be emphasized that the inflammatory response is sensitive to age (immunosenescence leads to a higher basal level of inflammatory markers) [31], sex (women generally have milder neuroinflammatory responses after TBI compared with males), and prior brain injuries (it is believed that a brain injury might ‘prime’ microglia into a more active state influencing the inflammatory response) [32].

Studies of mTBI have been limited to measuring inflammatory markers in less invasive fluid compartments such as blood. However, the concentration of blood-based inflammatory markers is much lower than concentrations identified in the cerebrospinal fluid (CSF) [32].

Importantly, given that inflammatory-associated proteins are produced by cells throughout the body in response to any disease-causing cellular injury, they are not highly specific for TBI. Inflammatory markers may be better suited as part of a multiple biomarker panel, including markers of other pathophysiological processes post-TBI, also having potential for decisions regarding athletes’ return to play or for predicting neuropsychological outcomes following mTBI.

#### 3.2.5. Other Markers

Extracellular vesicles (EVs) are emerging as biomarkers, as they can be secreted from all types of brain cells and exhibit specific markers on their surface. Intraluminal DNA, RNA, protein, and metabolites are indicators of the state of the cell of origin. The major pitfall in using EVs as biomarkers is their isolation: the presence of other components of biological fluid, including lipoproteins, chylomicrons, and microvesicles, interferes with the isolation process, and this, together with EVs’ nanoscale size and difficulties in separating particular sub-types, makes isolation a very challenging process [11].

MicroRNA abnormalities are also relevant to many neurodegenerative diseases and brain injuries such as TBI. Dysregulated levels correlate with impaired memory, learning, cognition, and neuropsychiatric disorders [33]. However, the current limitation to the use of miRNA biomarkers is their variable expression between different individuals; this high heterogeneity makes it difficult to determine the optimal cut-off values for using miRNA biomarkers for TBI diagnosis and prognosis [11].

Exosomes and miRNAs have recently gained considerable attention as promising biomarkers for TBI. However, despite the current knowledge of their potential, these biomarkers have not yet been optimized for clinical practice.

### 3.3. Methodological Assessment of mTBI Biomarkers

**Figure 2:**
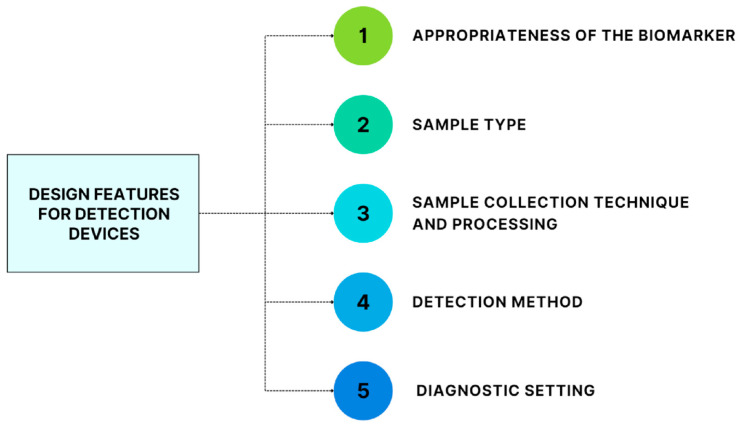
Design features to consider when developing mTBI detecting devices or laboratory tests.

There are several features to consider when designing platforms for detecting TBI biofluid protein biomarkers: the appropriateness of the biomarker, the type of samplee to use, the techniques of sample collection and processing, the choice of detection method, and the diagnostic setting in which the platform will be used (Figure 2).

(1) Appropriateness of the biomarker. The measured biomarkers should already provide proven clinically actionable information and be approved by regulatory agencies [34].
(2) Sample type. Protein biomarkers can be found in CSF, serum, and plasma [34], as reported in depth for each biomarker in the previous paragraph.
(3) Sample-collection technique and processing. They are conditioned by the choice of sample type, dictating kinetics, bioavailability, and assay detection limit [35].
(4) Choice of the detection method. Conventional clinical immunoassays are antibody-based methods that exploit automated platforms based on turbidimetry or nephelometry. They are spectrophotometric-based methods, and rely on the formation of an immune complex that scatters light; they can suffer reduced signal-to-noise levels in samples with high protein or lipid concentrations (such as serum) that cause non-specific light scattering. Other antibody-based detection methods are based on antibodies conjugated to various labels such as enzymes, fluorophores, and chemiluminescent compounds (as in enzyme-linked immunosorbent assay (ELISA), sandwich ELISA, and competitive ELISA). Most immunoassay-based methods currently available in the clinical laboratory analyze each target of interest independently; therefore, analysis using a panel of biomarkers would require separate aliquots of the sample [34,36]. On the contrary, multiplexed assays allow the simultaneous measurement of multiple analytes, and are therefore more suitable for detecting a panel of protein biomarkers for mTBI. Multiplexed assays can be divided into planar and microsphere-suspension designs. The planar immunoassays are similar to traditional single-target immunoassays. They involve different microspots with specific capture antibodies arranged in a two-dimensional layout: The capture antibodies bind to the target, there is the addition of a detection antibody, and the specificity of the signal is then indicated by the x,y location. Microsphere-suspension immunoassays are most frequently used for multiplexed antibody-based assays: the capture antibodies are bound to microspheres that can be univocally identified by size or fluorescence, the microspheres bind to the target antigen, followed by the addition of a labelled detection antibody, and the beads are then determined by flow cytometry [36,37]. In the mTBI context, multiplexed immunoassays are preferred as they allow simultaneous measurement of multiple analytes with reduced sample volume. Their use is not without limitations: They require a comprehensive method of validation and complex quality control procedures. There can also be further complications if one or more quality control results fail for some but not for all the analytes, and it can be challenging to optimize the analytical conditions for several antigen–antibody interactions [34,36].
(5) The diagnostic setting. In the context of mTBI, it would be ideal to have a device that could be used inside and outside the hospital. A point-of-care (POC) platform would enable rapid triage directly at the site of injury without requiring processing in a laboratory, thanks to its small and portable nature, the reduced requirement of sample volume, and fast turnaround time. It could also be used for monitoring and assessment in the ambulance and for monitoring the patient’s response at the hospital, with repeated measurements [32,36,38]. Many POC designs are available, but a critical difference that can enhance their quality is the presence of a built-in control that can indicate the correct execution of the assay. The most relevant POC designs in the mTBI context are lateral flow and cartridge/cassette devices. The detection they provide can be based on different principles (visual observation by the operator, charge-coupled device cameras, absorbance, surface plasmon resonance, fixed-polarized ellipsometry, diffraction, etc.) [36].

The ideal device for measuring TBI protein biomarkers would be able to quickly measure a panel of biomarkers with high sensitivity and specificity, requiring only a small sample volume and minimal sample preparation to promptly measure different biomarkers [39].

### 3.4. Technological Development Stage of Biomarker Detection Devices

**Figure 3:**
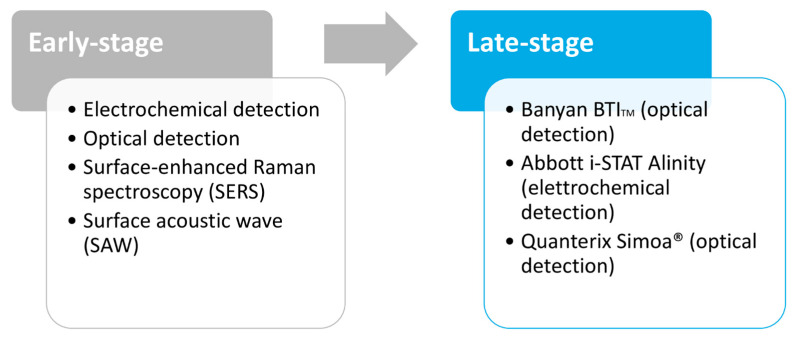
Current stage of development of detection devices for fluid biomarkers.

The devices are categorized based on their current stage of development: early-stage devices used in academia and late-stage devices commercially available (see Figure 3).

The measurement methods of early-stage devices are based on different detection methods: electrochemical detection, optical detection, surface-enhanced Raman spectroscopy (SERS), and surface acoustic wave (SAW) [39].

Electrochemical sensors use a three-electrode system (counter, working, and reference electrodes) to convert chemical changes into electrical signals. In the case of mTBI biomarkers, the working electrode is functionalized with antibodies for the biomarker of interest or with an enzyme that converts the target biomarker into an electrically detectable product, and the signal is measured using impedimetric, amperometric, or potentiometric techniques. This technology has already been used to quantify GFAP, NSE, S100B, and tumour necrosis factor-α (TNF-α) [40,41].

Optical detection quantifies analytes using techniques such as fluorescence, chemiluminescence, and colourimetry, and it is commonly used in sandwich ELISA immunoassays [42]. For TBI biomarkers, the binding reaction can occur on a chip or in a centrifuge tube, and there is the possibility of multiplexing, as developed by Krausz et al. for GFAP, interleukin-6 (IL-6), and interleukin-8 (IL-8) [43].

Surface-enhanced Raman spectroscopy (SERS) biosensors are based on inelastic light scattering. When the molecules are adsorbed to metal surfaces, such as gold nanoparticles, the scattering is enhanced, allowing the rapid and label-free detection of the analytes of interest [44,45]. A critical limitation in developing SERS-based POC is that any imperfection on the SERS substrate significantly reduces the analytical performance [46]. SERS-based detection has been incorporated in lateral flow assays to measure GFAP, NSE, and S100B [47,48,49].

Surface acoustic wave (SAW) biosensors detect frequency changes in an acoustic wave travelling along a piezoelectric crystal surface [50]. They can detect GFAP on a chip by functionalizing the SAW resonator with antibodies for GFAP; the binding with GFAP then causes a shift in the resonance frequency that is proportional to GFAP concentration [51].

Among all the sensors reviewed, electrochemical sensors are the furthest advanced towards their use as POC in the field; these types of sensors are also already in use as monitoring devices for blood glucose, so there is a precedent for their use for assessment in the field [52]. Optical sensors are widely used in laboratory settings. The difficulty is developing small and robust devices that can incorporate all the reagents and washing steps on a chip for use as POC. SERS could be attractive for TBI assessment since they enable label-free detection. However, the substrates are fragile, and the optics are complex; therefore, developing a POC test based on SERS is difficult. Furthermore, SAW sensors cannot be developed into POC tests since they are expensive and unsuitable for use outside the laboratory environment. Further development is therefore required to produce a device that can be used both inside and outside the hospital [39].

Currently, three systems are already in use in clinical studies or clinical practice and can therefore be considered to be in late stages of development (see Table 2).

The Banyan Brain Trauma Indicator (BTITM) from Banyan Biomarkers is based on optical detection (chemiluminescent ELISA) and measures GFAP and UCH-L1 in human serum. FDA approved it in February 2018 to support management of mTBI patients: A negative result can rule out the need for a CT scan. The main limitation of this test is that it requires 2 h for the execution. It has been helpful in the study of GFAP and UCH-L1 kinetics, their diagnostic accuracy, and their association with CT results; however, timely result is fundamental for TBI clinical management. Furtherm ore, it does not allow multiplexing, and it involves different test kits for GFAP and UCH-L1 on separate 96-well plates [53]. In 2019, Banyan Biomarkers provided a non-exclusive license for their TBI biomarkers to Abbott for use with their core laboratory instruments [54].

The FDA approved the TBI plasma cartridge for the Abbott i-STAT Alinity in January 2021, based on the Banyan BTITM. It is a portable device that uses electrochemical detection (amperometry) to measure GFAP and UCH-L1 levels (in multiplexing) in an EDTA anticoagulated plasma sample of 20 µL, in 15 min. It is designed to be used by trained personnel in a clinical laboratory setting, and a positive result (cut-off values are 30 pg/mL for GFAP and 360 pg/mL for UCH-L1) identifies patients who require a CT scan [55]. The reportable range for GFAP is 30–10,000 pg/mL and for UCH-L1 is 200–3200 pg/mL, while the estimated lower limit of quantification is 23 pg/mL for GFAP and 70 pg/mL for UCH-L1 [56]. It received the CE mark in December 2021 and is currently available outside the USA. Currently, the i-STAT Alinity TBI cartridge requires an EDTA anticoagulated plasma sample (prepared by healthcare professionals in a clinical laboratory setting); implementing a test that works with a whole blood sample would allow its use in field settings since the device is already portable [57,58].

The Quanterix Simoa^®^ bead technology is a variety of sandwich ELISA based on the optical detection of fluorescence. It allows multiplexing by labelling the beads with different fluorescent signatures. Then, both the enzyme-generated fluorescence and the bead fluorescence are measured to determine the signal for each biomarker [59]. This platform can detect TBI biomarkers, including GFAP, UCH-L1, Tau, NF, and NSE. However, the analysis takes 2.5 h, and the platform is not yet approved by the FDA; therefore, these assays can be used only for research purposes [60,61].

**Table 2:** Summary of the features of current late-stage sensors for measuring TBI biomarkers.

Device	Detection Technique	Biomarkers	Sample	Analysis Time	Multiplex	References
Banyan BTI	Optical (chemiluminescence)	GFAP, UCH-L1	Human serum	>2 h	No	[54]
Abbott i-STAT Alinity	Electrochemical (amperometric)	GFAP, UCH-L1	Human plasma	15 min	Yes	[55,56,57,58]
Quanterix Simoa^®^	Optical (fluorescence)	GFAP, UCH-L1, Tau, NF, NSE	Human serum, plasma, and CSF	2 h 30 min per plate	Yes	[59,60,61]

## 4. Discussion

### 4.1. Impact of mTBI Biomarkers on the Diagnostic-Therapeutic Pathway

Of all the reviewed biomarkers, GFAP, UCH-L1, NF proteins and IL-6, mainly when used in combination, appear to be the most appropriate to support the clinical management of mTBI. The combination of GFAP and UCH-L1 performed better than either biomarker alone in predicting intracranial injuries on head CT after TBI, also outperforming S100B, NfL, and T-tau in predicting intracranial pathology on head CT or brain structural MRI [25]. The mTBI biomarkers could have an impact, particularly in the sports context, allowing a quick return to play for athletes who test negative for the biomarkers.

The United States Food and Drug Administration recently approved a combination panel of blood GFAP and UCH-L1 to predict the absence of intracranial injuries on head CT. These blood-based biomarkers are helpful diagnostic tools and may reduce the use of CT scans in the emergency department setting and sports-related concussions on the field [25]. To date, clinical usefulness is restricted to identifying patients with a low risk of intracranial injury, reducing the number of patients that undergo head CT scan.

Extensive international collaborations, including the CENTER-TBI [62] and TRACK-TBI [63], represent encouraging research into biomarkers that can predict outcomes or monitor disease progression in the medical clinic [32]. The correct combination of biomarkers might create highly sensitive and specific combination panels. Inflammatory markers, combined with biomarkers that reflect cellular, glial, and axonal damage, may play an essential role in the formation of these panels and the development of POC devices that could enable the diagnosis of TBI at the scene, being especially relevant in sports settings, improving decisions regarding return to play for athletes and predictions of neuropsychological outcomes following mTBI.

### 4.2. Analytical Performances and Clinical Reliability

Herein, the performance of each biomarker for detecting intracranial lesions on CT is reviewed, reporting their sensitivity, specificity, and positive and negative predictive values with 95% confidence intervals to detect (or predict) all mTBI (complicated and uncomplicated) versus no mTBI. Receiver operating characteristic (ROC) curves were used to assess the ability of the biomarkers to distinguish between injured and uninjured control participants; for each biomarker, the area under the curve (AUC) is reported (AUC = 0.5 indicates no discrimination, AUC = 1.0 indicates a perfect diagnostic test), together with the ideal detection time. All results are summarized in Table 3.

GFAP can be measured in blood and CSF [25] at 20–24 h post-injury; it is highly specific for CNS [25]. The serum concentration was analyzed using the enzyme-linked immunosorbent assay platform from Banyan Biomarkers Inc. The area under the receiver operating characteristic curve (ROC), which measures test accuracy, ranged from 0.74 to 0.98, indicating good to excellent discrimination for predicting CT abnormalities [64,65,66].

Moreover, a recent study highlighted that serum GFAP levels had a slightly greater discriminability than plasma GFAP levels for detecting intracranial lesions on head CT: Serum GFAP had an AUC of 0.814, whereas plasma GFAP had 0.778 [67]. In Lewis et al. [68], classification performance was assessed by the sensitivity, specificity, and positive and negative predictive values with 95% confidence intervals (CIs) of a single serum concentration of GFAP within 6 h of head injury. GFAP classified mTBI versus no mTBI with an AUC of 0.70 (95% CI = 0.64–0.77). A cut-off level of 30 pg/mL GFAP yielded a sensitivity of 44.2% (36.9–51.6) and a specificity of 94.9% (85.9–98.9). NPV was 34.8% (27.5–42.7) and PPV 96.5% (90.1–99.3). GFAP had lower sensitivity than the other biomarkers but high specificity and PPV [68]. A single serum concentration of GFAP within 6 h of the head injury may help identify and stratify the severity of brain injury in emergency department patients with head trauma.

As GFAP, UCH-L1 can be measured in blood and CSF [25]. Its levels increase within the first 6–24 h after TBI, with an acute peak at ~8 h [11,25,69]. It is expressed in CNS, distal renal tubules, and islets of Langerhans [25]. In a study by Papa et al. [70], ROC curves were used to explore the ability of the biomarker to distinguish between injured and uninjured control participants and TBI patients within 4 h of injury, as well as for intracranial lesions on CT scan. The area under the curve (AUC) was calculated from the ROC curves to assess the performance of early UCH-L1 levels in distinguishing TBI from control patients. Early UCH-L1 levels were able to distinguish TBI from uninjured control participants with an AUC 0.87 (95% CI 0.82–0.92), indicating good predictive accuracy [70,71]. The area under the curve for discriminating between positive and negative intracranial lesions on CT scan was 0.73 (95% CI 0.62–0.83) [70].

The classification performance was assessed by sensitivity, specificity, and positive and negative predictive values with 95% confidence intervals (CIs). A cut-off level of 30 pg/mL yielded a sensitivity of 78.2% (95% CI 72.3–83.5), a specificity of 37.3% (95%CI 25.4–49.2), a positive predictive value of 79.9% (95% CI 76.6–83.3), and a negative predictive value of 34.9% (95% CI 25.0–45.5) [68]. However, different cut-offs provided different results. For instance, a cut-off of 0.09 ng/mL yielded a sensitivity of 100% (95% CI 88–100), a specificity of 21% (95% CI 13–32), and a negative predictive value of 100% (76–100) [70]. UCH-L1 alone may lack suitable specificity; some findings have shown that the biomarker failed to differentiate between mTBI patients and orthopaedic controls, and its inclusion in panels of multiple biomarkers is recommended to achieve higher accuracy [72].

The Abbott i-STAT Alinity TBI cartridge measures the combination of GFAP and UCH-L1; these were the first biomarkers to be cleared in 2018 by the US FDA to help determine the need for CT scans in mild to moderate TBI adult patients (within 12 h of injury). The FDA approval was based on results from the ALERT TBI multicenter trial (2012–2014) that demonstrated that UCH-L1 and GFAP measurements (cut-off values of 327 pg/mL for UCH-L1 and 22 pg/mL for GFAP) together showed a high sensitivity of 97.6% (95% CI 0.931–0.995) and an NPV of 99.6 (95% CI 0.987–0.999), therefore allowing the exclusion of patients in ED. The assessed specificity was 36.4% (95% CI 0.342–0.387) and the PPV was 9.5% (95% CI 0.079–0.112) [73].

Similar findings were observed in a TRACK-TBI multicenter observational study; the individual AUC for GFAP and UCHL1 were respectively 0.91 and 0.87, but the combination of the two markers yielded an AUC of 0.94 for discriminating between TBI patients and healthy controls and an AUC of 0.88 for distinguishing between CT+ and CT− TBI cases [58,74,75].

Neurofilament proteins (NFs) represent another promising blood biomarker for mTBI, and can be measured in blood and CSF [25]. They are mainly specific to the brain; they can also be found in the peripheral nervous system since they are released following axonal injury [12,25]. The temporal profile of NF differs from the other blood biomarkers; NFs levels rise throughout the first 12 days post-injury and correlate with CT lesions, predicting outcomes 12 months after TBI [11,12]. In particular, NF-L levels can distinguish patients with TBI from controls up to six months post-injury and between patients with mild, moderate, and severe TBI at 30 d post-injury. Elevated levels were also associated with reduced volumes of grey and white matter and alterations to white matter integrity [24,76].

Using single-molecule array technology and a cut-off of 24.0 pg/mL, Shahim et al. [77] demonstrated that NF-L quantification at admission yielded an AUC of 0.99 for detecting TBI versus controls, which increased to 1.00 at day 12. Applying these cut-off levels yielded a sensitivity of 97% and a specificity of 96%. The positive likelihood ratio (LR+) was 23.0 for NF-L, although the positive and negative predictive values were not assessed [77]. Accordingly, NF-L concentration is a sensitive and reliable biomarker for repetitive and subconcussive head impacts in both laboratory and field settings; its specificity to neuronal axons and strong associations with TBI severity and outcome make NF-L a very promising biomarker for brain injury [11,24].

**Table 3:** Analytical features of each TBI biomarker.

Marker	Sample	Detection Time	Cut-Off	CNS Specificity	Sensitivity %	Specificity %	NPV %	PPV %	AUC	Method	Refs.
GFAP	Blood/Serum and CSF	20–24 h post-injury	30.0 pg/mL	Yes	44.2	94.9	34.8	96.5	0.74–0.98	ELISA platform (Banyan Biomarkers Inc.)	[25,64,66,68]
UCH-L1	Blood/Serum and CSF	6–24 h post-injury	30.0 pg/mL	Also expressed in distal renal tubules and islets of Langerhans	78.2	37.3	34.9	79.9	0.87	ELISA platform (Banyan Biomarkers Inc.)	[68,70,71]
NFs	Blood and CSF	Rise through 12 d post-injury	24.0 pg/mL	Also found in PNS	97	96	Not reported	Not reported	0.99	ELISA single-molecule array technology for quantification of NF-L	[77]
IL6	Plasma or serum	6–8 h post-injury	Not reported	No	Not reported	Not reported	Not reported	Not reported	0.81	Ultrasensitive single-molecule ELISA (SIMOA)	[30]
IL6	Serum	6–8 h post-injury	Not reported	No	52	81	Not reported	Not reported	Not reported	MSD V-PLEX assays	[29]
GFAP + UCH-L1	EDTA anticoagulated plasma sample (20 µL)	Within 12 h of injury	22 pg/mL GFAP and 327 pg/mL UCH-L1	Yes	97.6	36.4	99.6	9.5	0.94	Abbott i-STAT Alinity	[70,73]

Interleukin-6 (IL-6) is the final significant biomarker to be included in combination panels to support the clinical management of mTBI. Serum IL-6 concentration increases within the first 6–8 h, and acute levels are associated with both MRI and CT findings and more protracted duration of symptoms after mTBI; after 24–48 h, IL-6 levels are again similar to control groups [12]. A study from Visser et al. [32] highlighted a different temporal profile of IL-6 in sports-related concussion (SRC) and military concussion versus the general unselected population with mTBI; in athletes and military personnel, there was an acute elevation (<8 h) followed by a return to baseline within 48 h, whereas the general unselected population showed alterations lasting up to six months after injury. A possible explanation could be that the inflammatory response is milder in the relatively young and healthy athlete population compared to the average mTBI patient [28,32]. Since inflammatory-associated proteins are produced by cells throughout the body, IL-6 is not specific for CNS injuries, and extracranial factors may influence its levels [12]. It is, therefore, recommended to include IL-6 in a multiple biomarker panel, together with markers of other pathophysiological processes post-TBI, for it to be helpful as an mTBI biomarker. Edwards et al. [30,78] determined the ability of IL-6 to differentiate concussed and healthy control subjects, by obtaining model ROC curves and performing area under the curve (AUC) analysis; IL-6 was a significant predictor and had an AUC value of 0.81 (95% CI 0.72–0.90) [30,78]. Meier et al. [29] estimated a sensitivity of 0.52 (standard deviation 0.17) and a specificity of 0.81 (standard deviation 0.11) by measuring IL-6 serum concentrations with a Meso Scale Discovery (MSD) QuickPlex SQ 120 instrument (MSD V-PLEX assays); the AUC was 0.75 (0.64–0.85) [29]. These two studies were based on comparing the baseline characteristics of healthy and concussed groups at different time points; they evaluated whether there was a significant difference in time-based change of IL-6 between the concussed group and the healthy control group, without using any cut-off value. Our literature search found no study reporting the positive and negative predictive value of IL-6 for the TBI context.

### 4.3. Analytical Limitations and Conclusions

This review of the current literature aims to provide both a comprehensive summary of the status of research into brain-injury blood biomarkers, and insight into the current guidelines and the characteristics of each biomarker. UCH-L1, GFAP, NF proteins, and markers of inflammation have shown high potential in distinguishing patients with trauma-related cranial CT findings from those without. Several recent studies have highlighted that a panel of biomarkers may outperform individual biomarkers, especially in terms of diagnostic and predictive value. A panel with UCH-L1, GFAP, NF proteins, and markers of inflammation could therefore show high utility if introduced in routine clinical practice to diagnose TBI, improve its management, and establish a reliable prognosis soon after TBI. Neuroimaging by CT and MRI is limited to detecting evident head injuries, with high cost, missing minute neural injuries or structural changes. On the contrary, blood biomarkers could enable a reliable and accurate correlation with neuronal, axonal, or glial cell injuries.

However, some issues are worth considering. A broad array of neurobiopsychosocial factors should be taken into account when using biomarker data at the system level. Age, biological sex, socioeconomic status, pre-existing conditions/comorbidities, comedications, etc., are essential factors that affect both the acute response to injury and the outcome [12]. Their inclusion increases the heterogeneity and complexity of mTBI cases, but will increase outcome prediction and help to design evidence-based, personalized treatments.

Furthermore, a detailed understanding of how comorbidities affect blood biomarkers measurements remains lacking, along with the expected values of biomarkers across different age groups. For these reasons, it would be easier to first introduce the clinical use of blood mTBI biomarkers in sports-related contexts. Athletes are generally young and healthy and would therefore provide a good starting sample, with no loss of specificity due to other comorbidities or related to age.

One of the most critical limitations affecting TBI management is the lack of a standard criterion for diagnosing and managing concussion. Each country has its own rules, and there is a need to establish unique and reliable European guidelines to improve the evaluation and management of mTBI patients in acute settings.

Our review also reveals the need for standardized methods of biomarker measurement. Current evidence is confounded by heterogeneous study design, analysis, and reporting; each study used different detection methods, cutoff values, and statistical analysis, yielding different sensitivities, specificities, VPP, and VPN. The results are concordant in highlighting the potential of blood biomarkers in improving mTBI management; however, their introduction in clinical use will require more rigorous assessment that will enable comparison between results and their validation.

In acute care settings, developing a clinically validated brain biomarker test can substantially alter diagnostic approaches to patients with TBI, aiding head CT decision-making in busy EDs, where the long wait for imaging contributes to overcrowding and reduced patient throughput. In the sports context, the prognostic role of mTBI biomarkers can allow on-the-field assessments of TBI, guiding the decisions regarding athletes’ return to play and reducing the occurrence of second-impact syndrome and repeated mTBI injuries. Particularly in the sports context, the data harmonization process is fundamental since professional athletes compete in different countries worldwide and may therefore need to take tests in other hospitals and countries.

When designing devices for detecting mTBI biomarkers, as shown in Figure 2, development of biomarker-measurement devices and clinical validation of protein biomarkers must co-occur. The sample type and its collection and processing techniques determine the diagnostic setting in which the device should be used. For instance, the FDA-approved Abbott iSTAT Alinity platform requires sample processing and does not work with whole blood; therefore, its process can be executed only in hospitals with specialized laboratories. An ideal device would be portable and usable by untrained personnel in non-ideal conditions (e.g., extreme conditions of temperature and humidity), requiring a small sample volume and a minimal sample preparation (ideally a drop of whole capillary blood obtained via a fingerstick) to quickly measure a panel of biomarkers with simultaneously high sensitivity and specificity. Furthermore, reagents should be stable, and manufacturing should be inexpensive to enable mass production of the device.

Hence, much must be considered before introducing tests for mTBI blood biomarkers into clinical practice. Still, they have the potential to improve diagnostic accuracy, predict the rate and severity of injury progression, and guide injury management, with a particular relevance especially in sports-related settings.

## 5. Future Directions

In the future, an update of the reference guidelines for TBI management is expected, including biomarkers to reduce the use of CT scans in the emergency department setting and to improve predictions of neuropsychological outcomes following mTBI.

In the sports context, the prognostic role of TBI biomarkers will allow on-the-field assessments of TBI, acting as a companion diagnostic for return to play and reducing the occurrence of second-impact syndrome and repeated mTBI injuries.

Besides their use in the sports context, the introduction of blood-based TBI biomarkers will also have a substantial impact in the military field, which, similarly to the sports context, requires immediacy and accuracy of detection and enables to start from a good sample of healthy subjects without other comorbidities.

## Figures and Tables

**Table 1:** Comparison between the already-in-use prediction rules for traumatic brain injury. NICE: National Institute for Health and Care Excellence; NCWFS: Neurotraumatology Committee of the World Federation of Neurosurgical Societies. High risk: risk factor is present in the prediction rule as a major criterion; medium risk: risk factor is present in the prediction rule as a minor criterion; blank: the variable is not a risk factor in the model.

Risk Factor	New Orleans Criteria	Canadian CT Head Rule	NICE 2014	NCWFS	Scandinavian
Headache	High risk			High risk	Medium risk
Vomiting	High risk	High risk	High risk	High risk	Medium risk
Post-traumatic seizure	High risk		High risk		High risk
Intoxication (drug or alcohol)	High risk			High risk	
Persistent anterograde amnesia	High risk			High risk	
Age	High risk > 60 years	High risk > 65 years	High risk > 65 years		
Clinical signs of skull fracture	High risk	High risk	High risk	High risk	High risk
Contusion of the skull	High risk	High risk	High risk	High risk	High risk
Signs official fracture	High risk				
Contusion of the face	High risk				
GCS score deterioration		High risk	High risk		High risk
Pedestrian versus vehicle		Medium risk	High risk		
Ejected from vehicle		Medium risk	High risk		
Fall from height		Medium risk	High risk		
Prolonged post-traumatic amnesia		Medium risk	High risk	High risk	Medium risk
GCS < 15 at presentation			High risk	High risk	High risk
Loss of consciousness				High risk	Medium risk
Neurologic deficit			High risk	High risk	Medium risk
Anticoagulation therapy			High risk	High risk	High risk
High-energy trauma					
Multiple injuries					
Pre-traumatic seizure				High risk	
Unclear trauma mechanism					
Previous neurosurgery				High risk	
S100B ≥ 0.1 μg/L					Medium risk

## Data Availability

Not applicable.
